# PREVALENCE AND TYPES OF PATIENT PORTAL USE AMONG OLDER ADULTS RECEIVING HOME HEALTH CARE

**DOI:** 10.1093/geroni/igad104.0577

**Published:** 2023-12-21

**Authors:** Julia Burdgorf, Mingche Wu, Chanee Fabius

**Affiliations:** Center for Home Care Policy & Research, New York, New York, United States; Johns Hopkins University, Baltimore, Maryland, United States; Johns Hopkins University, Baltimore, Maryland, United States

## Abstract

Home health care (HHC) delivers skilled nursing and therapy services in the patient’s home and is a valuable source of care for community-living older adults, particularly those living with complex conditions such as dementia. However, lack of visibility into patients’ clinical records and difficulty communicating with other providers are perennial challenges to HHC quality. Patient portals― provider-sponsored online applications that allow patients to perform care management tasks including viewing health information and messaging providers―present a promising opportunity to bridge this gap. However, no prior work has examined patterns of portal use among HHC patients. We leveraged a unique dataset containing electronic health record and patient portal utilization data for 10,212 older adults who received HHC between 2017-2019. We found that 25% of HHC patients had an active patient portal prior to HHC and, of those with an active portal, 85% used their portal during HHC. Among those who used their portal during HHC, the most common portal activities were reviewing the clinical record (82%), managing appointments (60%), and seeking provider advice (45%). Compared to community-referred patients, hospital-referred patients were more likely to use their portal to manage appointments or review their clinical record. Compared to patients without dementia, patients with dementia were more likely to use their portal to message their provider but less likely to view their clinical record. Findings suggest that portal users are actively engaging with their portal during HHC, indicating an opportunity to empower patients to facilitate information transfer between HHC and other providers.

